# Traditional Medicine in Management of Type 2 Diabetes Mellitus

**DOI:** 10.1155/2013/580823

**Published:** 2013-07-24

**Authors:** Syed Ibrahim Rizvi, Elena Matteucci, Pinar Atukeren

**Affiliations:** ^1^Department of Biochemistry, University of Allahabad, Allahabad 211002, India; ^2^Department of Clinical and Experimental Medicine, Pisa University, 56126 Pisa, Italy; ^3^Department of Biochemistry, Cerrahpasa Medical School, Istanbul University, 34303 Istanbul, Turkey

The incidence of type 2 diabetes mellitus has now reached epidemic proportions. Although the disease manifests in the form of hyperglycemia, the cause could be varied ranging from disturbance in insulin secretion, insulin action, insulin resistance, glucose production and glucose uptake, interplay between different hormones, and various kind of stress. Due to such varied etiology, the management of diabetic condition poses a great medical challenge. No single agent has so far been unequivocally accepted as the antidiabetic drug.

Alternative systems of medicine based on traditional wisdom have thrived through ages and are still practiced by a large population for the management of diabetes. A large number of plants have proved their efficacy in management of diabetes especially hyperglycemia. In many cases, scientific studies have validated the antidiabetic nature of plant-based medicines, and the bioactive principle has been isolated and characterized. It is important that more research is done to understand the mechanism(s) involved in the antidiabetic action of large number of plant-based medicines used as traditional therapy for the management of diabetic condition.

The present special volume has brought together some interesting papers reporting the findings of the use of traditional medicines for the treatment of diabetes mellitus.

S. I. Rizvi and N. Mishra provide a good review of the anti-diabetic potential and the bioactive compounds present in *Ficus religiosa*, *Pterocarpus marsupium*, *Gymnema sylvestre*, *Allium sativum, Eugenia jambolana, Momordica charantia*, and *Trigonella foenum-graecum*. All these plants are widely used in the Indian subcontinent for the management of diabetic condition.

O. Eleazu et al. explore the chemical composition of cocoyam and unripe plantain flours and their potential in the dietary prevention of diabetic complications.

D. Bailbè and colleagues evaluate the effects of Subetta (containing release-active dilutions of antibodies to beta-subunit of insulin receptor and antibodies to endothelial nitric oxide synthase) in Goto Kakizaki diabetic rats and demonstrate that 28-day administration improves glucose control to an extent similar to that of Rosiglitazone. 

H. Y. Jin et al. have tested the efficacy of DA-9801, a mixture of extracts from *Dioscorea japonica* and *Dioscorea nipponica* in the treatment of diabetic peripheral neuropathy in experimental diabetes. They have also presented a comparison of the effect of DA-9801 with lipoic acid.

Statins are very widely used during dyslipidemia. Since type 2 diabetes is frequently associated with dyslipidemia, it is important to investigate the effect of statins on glucose homeostasis in diabetes. Wang et al. report that simvastatin may cause hyperglycemia and have an adverse effect on glucose homeostasis in diabetic rats.

*Syed Ibrahim Rizvi* *Syed Ibrahim Rizvi* *Elena Matteucci* *Elena Matteucci* *Pinar Atukeren* *Pinar Atukeren*

